# Magneto‐Controlled Tubular Liquid Actuators with Pore Engineering for Liquid Transport and Regulation

**DOI:** 10.1002/advs.202406325

**Published:** 2024-08-13

**Authors:** Huan Zhao, Ruyi Wen, Liyun Zhang, Linfeng Chen, Huizeng Li, Fan Xia, Yanlin Song

**Affiliations:** ^1^ State Key Laboratory of Biogeology and Environmental Geology, Engineering Research Center of Nano‐Geomaterials of Ministry of Education, Faculty of Materials Science and Chemistry China University of Geosciences Wuhan 430074 P. R. China; ^2^ Key Laboratory of Green Printing, CAS Research/Education Center for Excellence in Molecular Sciences, Institute of Chemistry Chinese Academy of Sciences (CAS) Beijing 100190 P. R. China

**Keywords:** liquid manipulation, liquid switch, magnetic field, polydimethylsiloxane (PDMS), pore structure, slippery surface, wetting

## Abstract

Liquid manipulation using tubular actuators finds diverse applications ranging from microfluidics, printing, liquid transfer to micro‐reactors. Achieving flexible and simple regulation of manipulated liquid droplets during transport is crucial for the tubular liquid actuators to perform complex and multiple functions, yet it remains challenging. Here, a facile tubular actuator for directional transport of various liquid droplets under the control of an externally applied magnetic field is presented. The surfaces of the actuator can be engineered with submillimeter‐sized through‐hole pores, which enables the liquid droplet to be easily modulated in the transport process. Furthermore, the liquid actuator with featured through‐hole pores is expanded to function as a switch in an integrated external electric circuit by magnetically controlling the motion of a conductive liquid droplet. This work develops a strategy for regulating liquid droplets in the tubular actuation systems, which may inspire ideas for designing functional liquid actuators with potential applications in microfluidics, microchemical reaction, liquid switch, and liquid robotics.

## Graphical abstract

To achieve flexible and simple regulation of liquid droplets in closed liquid transport systems, a new type of tubular liquid actuators with surface pore engineering is developed. Under the control of an external magnetic field, the liquid droplet in the actuator can be manipulated for movement, physical/chemical modulation, or serving as a switch in an integrated external electric circuit.

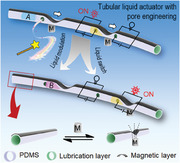

## 1 Introduction

Controlled liquid transport is fundamentally crucial in a wide range of applications from microfluidics,^[^ ^1^, ^2^, ^3^ ^]^ sensing,^[^ ^4^ ^]^ thermal management,^[^ ^5^ ^]^ printing,^[^ ^6^, ^7^, ^8^ ^]^ oil/water separation^[^ ^9^, ^10^ ^]^ to microchemical reaction,^[^ ^11^, ^12^ ^]^ and has drawn great attention in the past decades. To achieve the controlled motion of liquid droplets, various active and passive strategies have been well developed. For example, constructing surface wettability gradient^[^ ^13^, ^14^ ^]^ and asymmetric structure‐induced Laplace pressure gradient^[^ ^15^, ^16^ ^]^ are the most frequently used to direct the liquid transport. However, the aforementioned methods usually suffer from quite low transport velocity (several micrometers to a few millimeters per second) or relatively short distance (less than 10 mm) due to the presence of moving resistance,^[^ ^17^, ^18^ ^]^ which limit the practical applications. In the past decade, the peristome surface of *Nepenthes alata* provides continuous inspirations for the design of surfaces which exhibit impressively fast and continuous transport of liquids.^[^ ^19^, ^20^, ^21^ ^]^ But it works efficiently only to liquids with low surface tensions, and significant liquid loss is inevitable due to the superhydrophilic property.^[^ ^22^ ^]^ Alternatively, active strategies using external stimuli, such as light,^[^ ^23^, ^24^, ^25^ ^]^ electric field,^[^ ^19^, ^26^ ^]^ heat and magnetic field^[^ ^16^, ^27^, ^28^, ^29^ ^]^ have also been intensively investigated to achieve the directional liquid transport. Among the methods mentioned above, magnetic actuation of liquid transport has received particular interest due to its long‐range interaction, safety, contactless, reversible and easy control.^[^ ^11^, ^16^ ^]^

As a specific form, tubular liquid actuation systems (e.g., microfluidics) have attracted much attention, which is attributed to their unique properties of directional liquid transport, avoiding evaporation and contamination. For example, microfluidics have shown their great potential applications in functional particle preparation, bioanalysis, protein crystallization, and microchemical reactions.^[^ ^30^, ^31^, ^32^, ^33^ ^]^ In recent years, tubular liquid actuation systems with smart control attract increasing scientific interest,^[^ ^7^, ^16^, ^23^, ^34^ ^]^ which could overcome the limitation of externally tethered pump and achieve the liquid transport with remote control. For example, Yu et al. reported an interesting photo‐controlled liquid tubular actuator made of liquid crystal polymer. Upon exposure to 470 nm light with a gradient intensity, asymmetric deformation of the tubular actuator would happen, which generated a Laplace‐pressure driving force to propel the liquid slug.^[^ ^34^ ^]^ Recently, Gong et al. fabricated a kind of biomimetic magnetic actuator by 3D printing technology, which could be used for pumping liquid under the magnetic field control.^[^ ^35^ ^]^ Most recently, inspired by the gastrointestinal peristalsis for viscous chyme transport, we developed a near infrared (NIR) light‐controlled hydrogel actuator for the transport of highly viscous liquids.^[^ ^7^ ^]^ However, in traditional tubular liquid actuation systems, regulating liquid droplets during transport becomes challenging due to the closed system‐induced limitation of direct interaction.^[^ ^33^, ^37^ ^]^ This complexity becomes particularly problematic in the events of errors or issues arise, necessitating corrections to the droplets. In most cases, specific tubular structures with multiple connections are often designed to control the fluid flow and achieve the physical or chemical modulation of the liquid droplet (e.g., mixing, loading, chemical modification, or cell encapsulation), which significantly increase the structural complexity and limit their applications. Therefore, developing a versatile and simple tubular liquid actuation platform that allows for easy regulation of liquid droplets during the transport process is crucial for the liquid actuators to perform complex and multiple functions.

**Figure 1:**
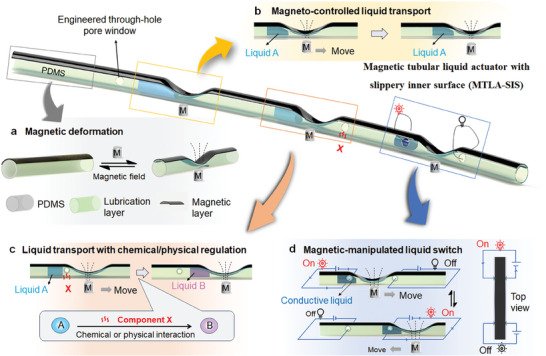
Schematic illustration of magnetic slippery tubular liquid actuator with engineered pore structures. a) The magnetic tubular liquid actuator with slippery inner surface (MTLA‐SIS) is made of a PDMS tube whose upper surface coated with a magnetic layer and inner surface infused with a lubricant layer. MTLA‐SIS could reversibly deform in response to an applied magnetic field. b) The MTLA‐SIS is able to propel liquid transport under the control of magnetic field. c) MTLA‐SIS for chemical/physical regulation of the liquid droplet in the transport process. With the pore window, liquid A in the MTLA‐SIS could be converted to liquid B. d) Magneto‐controlled liquid switch. Conducting liquid droplet could be manipulated to switch on/off the external electric circuits.

In this work, we design and develop a kind of magnetic tubular liquid actuator with slippery inner surface (MTLA‐SIS) for directional liquid transport (**Figure** **1**). The MTLA‐SIS is made of polydimethylsiloxane (PDMS) tube whose upper surface is coated with a thin layer of magnetic nanoparticles and inner surface is infused with lubricant (Figure 1a). The upper part of the tubular actuator is able to reversibly deform in response to an applied magnetic field, forming an asymmetric conical structure, which produces a Laplace pressure gradient to actuate liquid to move directionally (Figure 1b). In addition, the MTLA‐SIS could be engineered with submillimeter‐sized through‐hole pore structures, which not only could enable the liquid droplet to be chemically/physically regulated on demand during the transport process (e.g., liquid A is changed to be liquid B) (Figure 1c), but the liquid droplet could work as a switch to control the integrated electric circuit (Figure 1d). This work may provide some ideas for the design of tubular liquid actuation systems with advanced functions, which may find potential applications in microfluidics, liquid transfer, microchemical reactors, and liquid switches.

## 2 Results and Discussion

### 2.1 Fabrication of MTLA‐SIS

The MTLA‐SIS is fabricated by using a modified reported method,^[^ ^29^ ^]^ and the process is shown in Figure S1 (Supporting Information). The fabrication details can be found in the Experimental Section. Briefly, the PDMS tube was firstly prepared by using a 3.0 mm diameter Al rod (with rough structure on the surface) as a template. After removal of the template, PDMS tube was obtained and then coated on one side with magnetic materials by a contact coating method, followed by heat curing. Finally, the inner surface of as‐prepared magnetic PDMS tube was infused with oil lubricant (e.g., liquid paraffin) to produce MTLA‐SIS.

**Figure 2:**
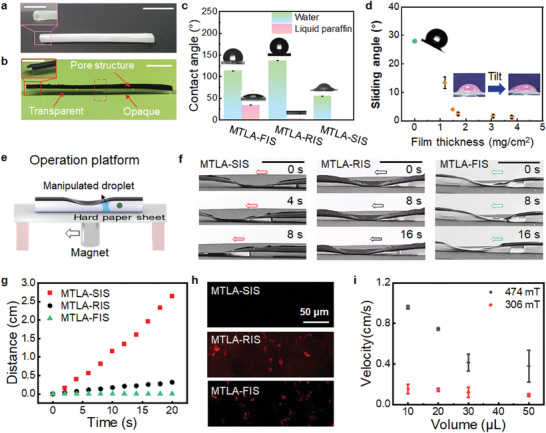
Structures and liquid actuation performance of magnetic tubular liquid actuators. a,b) Photographs of a PDMS tube with rough inner surface without and with magnetic coating. The inset is a cross‐sectional view of the corresponding samples. c) Contact angles of water and liquid paraffin on the inner surface of magnetic tubular PDMS with flat inner surface (MTLA‐FIS), rough inner surface (MTLA‐RIS) and slippery inner surface (MTLA‐SIS), respectively. d) Sliding angles of a water droplet (≈10 µL) on the inner surface of MTLA‐SIS with different amount of infused liquid paraffin. With the increase of the lubricant film thickness, the sliding angle dramatically decreases. As a control, the sliding angle on MTLA‐RIS (i.e., without the infused lubricant) is found to be ≈28°. e) Schematic illustration showing the magnetic control of liquid transport. f) Time‐lapse images showing the magnetic transport of water droplet by MTLA‐SIS, MTLA‐FIS, and MTLA‐RIS, respectively. g) Water transport distance versus time corresponding to the samples in (f). h) Fluorescence image of the inner surface of MTLA‐SIS, MTLA‐FIS, and MTLA‐RIS after magnetic transport of water droplet (containing Rhodamine B, 1 × 10^−3^ m). No fluorescence was observed on the surface of MTLA‐SIS. i) Water droplet transport velocity correlated with the magnetic intensity and liquid volume. The scale bar in (a), (b), and (f) is 1.0 cm.

The obtained PDMS tube was white in appearance due to the presence of the micro‐nano structures of the inner surface (**Figure** **2**a and Figure S2a,b, Supporting Information). After infused with liquid paraffin, it became transparent (Figure 2b), because oil lubricant decreased the difference in refractive index at the solid–air interface of these structured surfaces.^[^ ^22^ ^]^ Scanning electron microscopy (SEM) further showed the details of PDMS tube structure (Figure S2, Supporting Information). Its inner surface was rough. The thicknesses of the PDMS tube and the magnetic layer were about 60.2 µm and 58.2 µm, respectively. The thickness of both PDMS and the magnetic layer could be adjusted as desired, and further details are referred to our previous study.^[^ ^29^ ^]^

The rough inner surface of the magnetic PDMS tube (without infused oil) was hydrophobic for water (contact angle, CA ≈ 137°), and water could not be easily move (sliding angle, SA ≈ 28°) due to the strong contact angle hysteresis (Figure 2c,d), indicating a large sliding resistance. However, it was superoleophilic for oil (e.g., liquid paraffin) (Figure 2c). As soon as the lubricant oil contacted the rough surface, it spread rapidly to form a homogeneous thin liquid film. Water droplet on the oil‐infused surface could easily slide off. With increase of the oil film thickness, the SA of water dramatically decreased from ≈13° (1.2 mg cm^−2^) to ≈2.3° (1.7 mg cm^−2^), and slightly changed versus further increased oil film thickness (Figure 2d). The results indicated the ultralow along‐surface resistance of water motion on the oil film surface. Without otherwise stated, an oil infusion amount of about 2 mg cm^−2^ was used for the following experiments, which was sufficient to cover the inner surface and enabled liquid droplet to move easily.

### 2.2 Magnetic Control of Liquid Transport by MTLA‐SIS

The magnetic liquid transport performance of MTLA‐SIS was investigated. The operation platform is shown in Figure 2e. The MTLA‐SIS was fixed on a hard paper sheet, and a magnet (magnetic field intensity of ≈306 mT) was controlled to move directionally under the paper sheet. A water droplet (≈20 µL) inside the channel could be driven by the magnetic field to move correspondingly (Figure 2f and Movie S1, Supporting Information). As control experiments, the magnetic tubular actuators with rough inner surface (MTLA‐RIS) (i.e., without impregnated lubricant compared with MTLA‐SIS) and with flat inner surface (MTLA‐FIS) were failed to propel the droplet to move. The transport distance versus time was plotted, and the magnetic liquid transport of MTLA‐SIS was the most efficient (Figure 2g), which was attributed to the low along‐surface resistance of liquid transport at the presence of lubricant layer. The difference could also be clearly observed when the liquid droplets were driven by gravity to move downwards, as shown in Figure S3 (Supporting Information). In another experiment, water droplets containing rhodamine B (RhB, 1 × 10^−3^ m) was magnetically controlled to transport, and then the inner surface of the actuator was characterized by laser scanning confocal microscopy (LSCM). No fluorescence was only observed in MTLA‐SIS, indicating water droplet could be efficiently propelled to move without liquid residues on the inner surface (Figure 2h).

The liquid transport velocity was correlated with the liquid volume. With increase of liquid volume from 10 to 50 µL, the maximum transport velocity decreased (Figure 2i). In addition, by applying magnetic field with stronger intensity, the maximum transport velocity could be significantly enhanced, and ≈1.0 cm s^−1^ was obtained when the liquid volume was about 10 µL. Unless otherwise specified, magnetic field with intensity of about 474 mT was utilized in the following experiments. Furthermore, MTLA‐SIS exhibited stable mechanical property. After 1000 times of cyclic magnetic deformation, no obvious change was observed (Figure S4, Supporting Information). MTLA‐SIS is a universal liquid actuation platform and could be used for magnetic transport of various aqueous liquids and some ionic liquids, such as potassium hydrogen phthalate solution (KHC_8_H_4_O_4_), salt solution and ionic liquid 1‐butyl‐3‐methylimidazolium bis(trifluoromesulfonyl) imide ([BMIm] TNf_2_) (Figure S5a, Supporting Information).

The inner infused surface is important for the smooth magnetic liquid transport, as it significantly reduces the liquid transport resistance. Thus, to form a stable infused surface is vital for the development of MTLA‐SIS. According to previous studies, slippery lubricants are selected based on the considerations as follows:^[^ ^37^ ^]^ 1) Relatively a low surface tension. Liquids with low surface tension could easily spread on the rough surfaces, forming a homogeneous and stable liquid film.^[^ ^38^ ^]^ 2) Low vapor pressure (<1 Pa) in order to avoid evaporation loss. 3) Chemical inertness, so it could be used universally in diverse situations. In this work, besides liquid paraffin, we selected other representative liquids as the lubricants for constructing MTLA‐SIS, such as silicon oil, olive oil and ionic liquid 1‐butyl‐3‐methylimidazolium bis (trifluoromethylsulfonyl)imide ([BMIm]NTf_2_) (Table S1 and Figure S5, Supporting Information). It is worth noting that when [BMIm]NTf_2_ was selected, MTLA‐SIS could be used for magnetic transport of waterborne systems and some organic liquids (e.g., CH_2_I_2_) (Figure S5D, Supporting Information). We also showed that MTLA‐SIS could be controlled to propel the liquid droplet to transport along a tilted slope (Figure S6, Supporting Information).

### 2.3 Mechanism of Magnetic Liquid Transport by MTLA‐SIS

**Figure 3:**
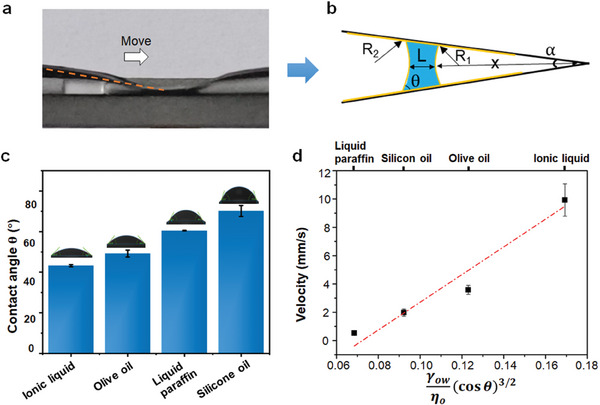
Mechanism of magnetic control of liquid transport. a) Snapshot of the magnetic liquid transport. b) A simplified model of the liquid droplet transport. c) Contact angles of water on the inner surface of the tubular liquid actuator infused with different lubricants. d) Experimental relationship between liquid droplet moving velocity and related factors.

The mechanism of the controlled liquid transport is attributed to the synergistic cooperation of asymmetric structure‐induced Laplace pressure gradient and lubricant layer‐induced low resistance. As shown in **Figure** **3**a, the magnetic field induced the deformation of PDMS tube, forming a wedgelike structure. Such an asymmetric geometry generates a capillary force to drive the water droplet to move towards the narrow end. A simple theoretical model is given in Figure 3b, and the Laplace pressure difference is derived as^[^ ^35^ ^]^

(1) $$\Delta p_{1}-\Delta p_{2}\approx \frac{4\gamma_{ow}L\cos\theta }{\alpha x\left(x+L\right)}$$

where the opening angle is denoted by *α*, the width of the liquid by *L*, the distance of the liquid from the apex by *x*, and the CA of liquid on the inner surface by *θ*. γ_*ow*_ is the interfacial tension between the water and oil lubricant. The contact angle hysteresis is negligible.

Here, two cases are considered, which are the droplet is relatively far from the apex (i.e., *x* ≫ L) and is close to the apex (*x* ≪ L). In the case of *x* ≫ L, the droplet is hardly propelled to move and the velocity is very slow (more details could be found in Scheme S1, Supporting Information). In the case of *x* ≪ L, the droplet could be well controlled to move fast, which is commonly shown in the paper. The driving force is obtained as^[^ ^39^ ^]^

(2) $$F_{d}\approx \frac{1}{3x}\pi \alpha \gamma_{ow}L^{2}\cos\theta$$

The viscous force resisting the motion of the droplet is given by^[^ ^40^ ^]^

(3) $$F\eta ∼\pi \alpha L\gamma_{ow}Ca^{2/3}$$

where *Ca* is the capillary number and is obtained by η_*o*_ *V*/γ_*ow*_, and η_*o*_ is the dynamic viscosity of the lubricant oil, and *V* is the moving velocity of the water droplet. In the case of the balance of the driving force and the resistance force, the maximum velocity could be obtained

(4) $$V∼{\left(\frac{L}{3x}\right)}^{\frac{3}{2}}\frac{\gamma_{ow}}{\eta_{o}}{\left(\cos\theta \right)}^{\frac{3}{2}}$$

According to the equation, the maximum droplet transport speed is linearly correlated with $\frac{\gamma_{ow}}{\eta_{o}}{\left(\cos\theta \right)}^{\frac{3}{2}}$. We performed magnetic water transport experiments (with magnetic intensify of 306 mT) with different liquid lubricants (water contact angles are different on the infused surfaces with different lubricants as shown in Figure 3c). The maximum water transport velocities were obtained when *x* was properly close to the apex and plotted with $\frac{\gamma_{ow}}{\eta_{o}}{\left(\cos\theta \right)}^{\frac{3}{2}}$ (Figure 3d). The experiment results are consistent with the equation (Figure 3d). The detailed analysis is referred to the Supporting Information.

### 2.4 MTLA‐SIS with Engineered Pores for Liquid Transport

**Figure 4:**
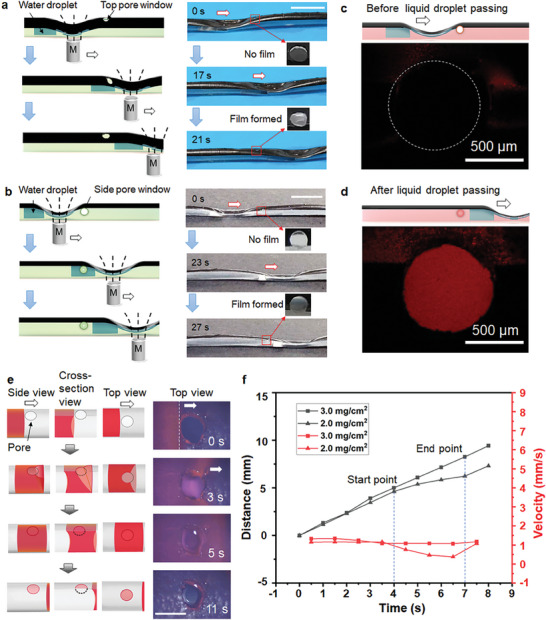
Magnetic transport of water droplets by MTLA‐SIS with engineered pore windows. a,b) Schematic illustration and experimental results of magnetic water transport by MTLA‐SIS with engineered pore window at the top surface and the side surface. The red arrows indicate the position of the pore window. c,d) Confocal laser scanning microscopy characterization of liquid film formation before and after magnetic control of droplet transport across the pore structure. e) Schematic and in situ experimental observation of the water droplet (≈20 µL) transport across the pore structure under gravity. f) Water droplet transport across the pore structure versus lubricant film thickness. The scale bar in (a), (b), and (e) is 1.0 cm.

As olive oil is more compatible with PDMS, we chose it as the lubricant in the following study. The MTLA‐SIS with submillimeter pore windows could be created by puncturing the actuator carefully using a blunt‐tip needle either on its side or magnetic coating surface, as depicted in Figure S1 (Supporting Information). The size of the pore can be adjusted by selecting a blunt‐tip needle of the desired diameter. As illustrated in **Figure** **4**a,b, MTLA‐SIS with an engineered pore window (≈0.75 mm in diameter) on the top or side surface was fabricated, respectively, and was demonstrated to actuate the directional transport of a water droplet (≈20 µL) under the control of an applied magnetic field. It could be seen that the water droplet could be magnetically guided to move directionally across an area with the pore window. Intriguingly, a liquid film was observed to form over the pore after the liquid passed through (Movies S2 and S3, Supporting Information). To confirm that the liquid film was formed by the oil lubricant or the water, we repeated the magnetic liquid transport process by adding a small amount of Nile red into the lubricant, and then observed it with CLSM. The pore window exhibited red fluorescence after the passage of the liquid droplet, indicating the liquid film was the lubricant oil (Figure 4c,d). In another experiment, we used calcein‐dyed water droplet. A dust‐free paper was controlled to contact the liquid film formed on the pore window, followed by CLSM observation. Only red fluorescence was again observed, which further confirmed the film was formed by the lubricant oil (Figure S7, Supporting Information).

Actually, the lubricant film formation process and stability were correlated with the lubricant layer thickness. In the case of the thickness larger than 2.0 mg cm^−2^, a relatively stable lubricant film easily formed when the pore experienced sequential magnetic deformation and recovery (before the liquid droplet moved across the pore window). Otherwise, the lubricant film was observed after the liquid droplet moved across the pore window. In order to confirm this point, we directly applied magnetic field to induce the pore deformation and observed whether the lubricant film formed after the magnetic field was removed (Figure S8, Supporting Information). In the case of lubricant layer thickness of 3.0 mg cm^−2^, we could observe the formed lubricant film (lasting tens of seconds) while no liquid film was observed when the lubricant layer thickness was 2.0 mg cm^−2^. In both cases, lubricant films were observed when a water droplet was magnetically controlled to move across the pore (Figure S9, Supporting Information). In addition, compared with the case of lubricant thickness of 3.0 mg cm^−2^, the formed film in the case of 2.0 mg cm^−2^ was rather thin, and the center area was difficult to be seen, which was not stable and quickly ruptured in the observation time (<15 s). In order to observe the film formation process in the case of 2.0 mg cm^−2^, we used CLSM to in situ observe the liquid droplet transport across the pore window. In this experiment, the water droplet was driven by gravity (as it was not easy to operate by applying magnetic field under the CLSM). As shown in Figure 4e, time‐lapse images showed the process of water droplet passing across the bottom pore window. Initially, the water droplet moved forward, and then the front line of the droplet was stopped when meeting the pore. Due to the presence of a high edge pinning effect,^[^ ^40^ ^]^ the water droplet moved continuously forward along the pore edge, resulting in the formation of an asymmetric curved surface. With the increase of the curvature, the edge barrier was finally overcome and a lubricant film was immediately formed on the pore (Figure 4e and Movie S4, Supporting Information).

As the lubricant film formation on the pore structure was dependent on the lubricant thickness, water droplet behaved differently correspondingly when passing across the pore. When the lubricant thickness was 2.0 mg cm^−2^, no lubricant film formed on the pore and the transport velocity of water droplet was slowed down due to the pore edge pinning effect (Figure 4f). In comparison, the water droplet moved smooth without obviously change when the lubricant thickness was 3.0 mg cm^−2^ as the lubricant film formed on the pore before the liquid droplet moved to the pore position, which significantly reduced the edge barrier (Figure 4f).

MTLA‐SIS could be magnetically controlled to propel the water droplet to move from right to left and reversibly from left to right (Figure S10, Supporting Information). No residue was observed in the inner surface of the actuator (Figure S11, Supporting Information). More importantly, no liquid leak happened when water droplet transported across the pore window (Figure S12, Supporting Information).

### 2.5 MTLA‐SIS for Liquid Transport and Regulation

Here, we demonstrated the proof‐of‐concept applications of MTLA‐SIS. The MTLA‐SIS with pore structures could be used for directional transport of various liquids under the control of an applied magnetic field (Figure S13a, Supporting Information). For example, MTLA‐SIS infused with olive oil was demonstrated to transport ionic liquids (e.g., [BMIm]NTf_2_), and commonly aqueous solutions, including salt solution, bovine whole blood, and fetal bovine serum (Figure S13b–e, Supporting Information). In addition, the inner surface of MTLA‐SIS could also be infused with non‐volatile ionic liquids, such as extensively studied [BMIm]NTf_2_,^[^ ^41^, ^42^, ^43^ ^]^ which would enable the liquid actuator not only to work under extreme conditions (e.g., in vacuum and relatively low temperature), but to propel both immiscible aqueous solutions (e.g., potassium hydrogen phthalate buffer, glycerin–water mixture, and fetal bovine serum) and organic solutions (e.g., CH_2_I_2_) (Figure S13f–i, Supporting Information). MTLA‐SIS could also be engineered with multipore structures, which was shown to be magnetically manipulated to control the water droplet transport forward and backward successfully (Figure S14, Supporting Information).

**Figure 5:**
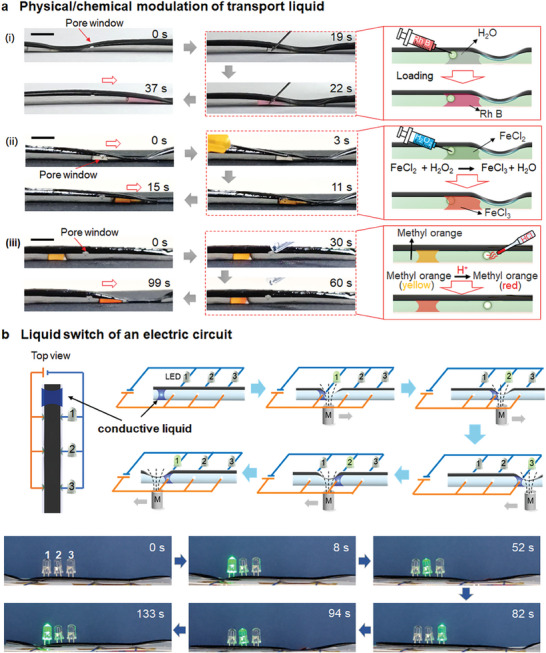
Demonstrations of MTLA‐SIS with pore windows for different functions. a) Physical and chemical regulation of the liquid droplet during the magnetic transport process. (i) A droplet of water is magnetically controlled to move to the position of the side pore window and changed to light red after introducing with rhodamine B solution. (ii) A droplet of FeCl_2_ solution (light green) is magnetically controlled to move to the position of the side pore window and changed to yellowish‐brown FeCl_3_ after reacting with H_2_O_2_. (iii) A droplet of methyl orange solution is magnetically manipulated to move and chemically modified by the reaction with HCl vapor. b) A liquid electric switch. Schematics and experimental illustration showing that the conductive liquid is controlled by MTLA‐SIS to sequentially switch on/off the LEDs. The scale bar is 1.0 cm.

In addition, MTLA‐SIS with pore windows enabled the liquid droplet to be physically or chemically regulated during its transport on demand. As shown in **Figure** **5**a(i), we demonstrated that a water droplet was manipulated to move to the pore position, and loaded with rhodamine B. We also selected a simple chemical reaction in solution to demonstrate the chemical regulation of the droplet [Figure 5a(ii)]. FeCl_2_ solution is able to react with H_2_O_2_, producing FeCl_3_. A droplet of FeCl_2_ solution in MTLA‐SIS was magnetically controlled to move to the pore window position. Then H_2_O_2_ was injected by a syringe through the pore to react with FeCl_2_ solution, resulting in color change from light green to yellowish‐brown. After the chemical modulation, the droplet was further manipulated to move forward. Liquid droplet could alternatively be chemically modulated via vapor contact. As a proof‐of‐concept, a droplet of methyl orange solution (≈20 µL) in the MTLA‐SIS was controlled to move to be near the pore window. HCl vapor diffused through the pore and reacted with methyl orange. After tens of seconds, the liquid droplet turned to be red from yellow [Figure 5a(iii)]. In other words, the liquid was successfully modified in the transport process.

Moreover, we demonstrated that the liquid droplet could function as a liquid switch for an external electric circuit under the control of magnetic field due to the presence of the pore structures. As shown in the schematics of Figure 5b, the MTLA‐SIS with three pairs of pores (≈0.75 mm in diameter) at the two side surfaces was fabricated and integrated to an external electric circuit loaded with three light‐emitting diodes (LEDs). Initially, the LEDs did not emit light because the electric circuit was not connected. Then 20 µL of conductive liquid (e.g., [BMIm]NTf_2_, or 1.0 m NaCl solution) in MTLA‐SIS was manipulated by an external magnetic field to move. Once the liquid droplet moved to the paired pore position, connecting the electric circuit, the corresponding LED would immediately emit green light. When the liquid was continuously controlled to move away from the paired pore position, the electric circuit was disconnected again and the LED would be switched off. In this way, LED 1, 2, and 3 could be controlled to be on or off (Figure 5b). The liquid electric switch is simple, safe, free from pollutants and could be remote‐controlled. In order to show the magnetic liquid actuator could be potentially used in sophisticated applications, we further designed and demonstrated magnetic liquid switches for sophisticated control of external circuits. Two magnetic tubular actuators, each featuring two pairs of through‐hole pores in each actuator (labeled A, B, C, and D), and were assembled and integrated with the external electric circuits (Table S2, Supporting Information). Specifically, pores A and B were connected in series with the left LED while pores C and D were connected in parallel with the right LED. The movement of conductive droplets was used to maneuver the pores, thereby establishing connections within the external circuits. A pore was designed as “1” When it was actuated by the droplet to make a connection; otherwise, it was “0”. Similarly, an LED was considered “1” when lit and “0” when off. A total of 16 possible outcomes could be obtained (shown in Table S2, Supporting Information).

## 3 Conclusion

In summary, we designed and developed a kind of PDMS‐based magnetic tubular actuator with designable pore structures for directional liquid transport and liquid regulation. Upon an externally applied magnetic field with predefined moving direction, the tubular actuator deformed correspondingly to form an asymmetric structure, which produced a driving force to propel the liquid transport. The oil‐infused inner surface facilitated the transport of liquid due to the ultralow resistance. The MTLA‐SIS with engineered submillimeter pore windows on its top or side surfaces was demonstrated to modulate the liquid droplet in the transport process. In addition, the MTLA‐SIS could be utilized to control the external electric circuit by manipulating the motion of conductive liquid droplets. This work provides insights into the design of closed tubular liquid actuators with advanced functions, which may find applications in microfluidics, microchemical reactions, liquid robotics, liquid electric switch, and information encoding. We acknowledge that the current study relies on manual magnet manipulation to achieve droplet motion, a method that may not be optimal for practical applications. Moving forward, we plan to develop an automated control system. This system will be integrated into our research to address the fundamental challenges of liquid manipulation more effectively. By automating the process, we aim to enhance the applicability and reliability of our approach.

## Experimental Section

A detailed description of experimental procedures can be found in the Supporting Information.

## Conflict of Interest

The authors declare no conflict of interest.

## Supporting information

Supporting Information

Supplemental Movie 1

Supplemental Movie 2

Supplemental Movie 3

Supplemental Movie 4

## Data Availability

The data that support the findings of this study are available in the supplementary material of this article.
