# Supplementary material for: Magneto‐Controlled Tubular Liquid Actuators with Pore Engineering for Liquid Transport and Regulation
Source: Adv Sci (Weinh). 2024 Aug 13;11(39):2406325. doi: 10.1002/advs.202406325 (PMC11497001; doi:10.1002/advs.202406325)

Supporting Information

Magneto-controlled tubular liquid actuators with pore engineering for liquid transport and regulation

Huan Zhao, Ruyi Wen, Liyun Zhang, Linfeng Chen*, Huizeng Li, Fan Xia*, Yanlin Song

Section 1 Experimental Section/Methods

1.1 Materials

Industrial Al (99+%, Bochuang metal materials company, China) rods (3 mm in diameter) were used as templates for tubular PDMS preparation. Hydrochloric acid (HCl), sodium hydroxide (NaOH), nitric acid (HNO_3_), sodium chloride (NaCl), hydrogen peroxide (H_2_O_2_), ferrous disulfide (FeCl_2_), methyl orange, glycerol, diiofomethane (CH_2_I_2_) and Rhodamine B (RhB) were purchased from Sinopharm (China). PDMS (Sylgard184, monomer and curing agent) was purchased from Dow Corning. Iron oxide nanoparticles (Fe_3_O_4_, 30-50nm), 1-butyl-3-methylImidazolium bis (trifluoromethylsulfonyl)imide ([BMIm]NTf_2_), silicon oil, liquid paraffin, potassium hydrogen phthalate (KHC_8_H_4_O_4_), and Nile red were purchased from Aladdin. Olive was obtained from Yuanye Bio-Technology Shanghai, China. Calcein was originated from InnoChem, Beijing, China. Fetal bovine serum was purchased from TianHang Biotechnology, Zhejiang, China. Bovine whole blood was obtained from Bersee, Beijing, China. The cylindrical magnets were purchased from Yiwu Hualuo, Zhejiang, China, with magnetic intensity on the top surface of 306 mT and 474 mT were 5 mm (diameter) x 2 cm (length) and 6 mm (diameter) x 2.5 cm (length), respectively.

1.2 Characterization

Scanning electron microscopy (SEM) images were collected with a field-emission scanning electron microscope (SU8010, Hitachi, Japan). Samples were coated with a thin layer of gold prior to SEM examination, which was performed on a magnetron ion sputter metal coating device (MSP-2S, Japan). The sliding angles (SA) and contact angles (CA) of different surfaces were measured with a contact angle goniometer (KRÜSS DSA100, Germany). The fluorescence images were obtained with a laser scanning confocal microscopy (LSM880 confocal microscopy, Carl Zeiss AG) equipped with a FemtoSecond Laser (Coherent Inc.). The fluorescence spectrophotometer (F-7000, Hitachi, Japan) was used to prove the liquids were not contaminated in the transport process. The rotational viscometer (NDJ-5S, China) was used to measure the viscosity of lubricants. The transport process of liquid droplets in the magnetic tubular actuator was recorded with a mobile telephone camera.

A detailed description of experimental procedures can be found in the Supporting Information.

**1.3 Preparation of magnetic tubular liquid actuators with slippery inner surface** Magnetic tubular liquid actuators with slippery inner surface (MTLA-SIS) were fabricated using a modified methods reported previously.^[29]^ As shown in Figure S2, Firstly, Al rod templates were immersed in NaOH solution (0.5 M) for 5 min to remove the oxide layer. Then, the Al rods were etched by HCl solution (2.5 M) for 30 min to produce microstructures, followed by washing with ultrapure water completely and drying. Subsequently, a mixture of PDMS monomer and curing agent (weight ratio 10:1) was prepared and degassed under vacuum for 20 min. Then, Al molds were vertically inserted into the PDMS prepolymer, followed by vacuum pumping for about 2 h. Al rods coated with PDMS prepolymer were vertically positioned for 20 min at room temperature to tune the thickness of the PDMS coating, followed by curing in an oven at 90 °C for 3 h. PDMS tubes were finally obtained by sequentially removing the Al templates with acid etching and washing completely with ultrapure water. To coat the PDMS tubes with magnetic coating, the mixture of PDMS prepolymer (monomer to initiator weight ratio 10:1) and iron oxide (Fe_3_O_4_) nanoparticles (55 wt%) was first coated onto a clean plastic plate by the blade coating process. The thickness was controlled by using a piece of cover slide (~0.13 mm in thickness) as a spacer. Then, the as-prepared PDMS tubes were controlled to contact with the magnetic PDMS prepolymer for coating. Subsequently, the PDMS tubes coated with the magnetic layer were placed in an oven for curing at 70°C for 4 h to finally give the magnetic PDMS tubular actuators (Figure S2). After filling the inner surface of the tubular actuators with a certain amount of lubricant, we obtained the MTLA-SIS. Alternatively, the magnetic PDMS tubular actuators could be engineered with sub-millimeter sized pore structure with a blunt-tip needle at the side or top surface, followed by adding with lubricant.

Magnetic tubular actuators with flat inner surface were also fabricated as the control by using PDMS tubes without rough inner surface structures, which were prepared with the procedures as described above, except that Al rods without rough structures were used as the templates. Unless otherwise specified, all other experiments were completed at room temperature.

**1.4 Lubricant dosage test**

Planar PDMS samples (~1 cm × 1 cm) were obtained by cutting the as-prepared PDMS tubes with rough inner surface open and fixed on glass slides with double-sided tapes. Then liquid paraffin of different qualities was infused into the planar PDMS surface. The glass slide was fixed on the platform of a contact angle goniometer (DSA 100). Subsequently, a water droplet (~10 μL) was placed on the PDMS surface, and the sliding angle was measured by tilting the platform slowly (rotation rate 90°/min) until the water droplet slid downwards. Each sample was measured 3 times. Planar PDMS sample without infused liquid paraffin was tested for comparison.

**1.5 Magnetic control of liquid transport**

The MTLA-SIS was fixed on a piece of paper (~0.22 mm thick) with double-sided tape, which was relatively hard to support the actuator. A cylindrical magnet with a surface magnetic intensity of approximate 306 mT or 474 mT was placed at the bottom of the paper and moved forth and back along the longitudinal direction of the actuator with manually controlled moving velocity (Figure 2e). A liquid droplet was added into the tubular actuator by a pipette, and was propelled to move under the control of the cylindrical magnet. Magnetic tubular liquid actuator with rough inner surface (MTLA-RIS) and with flat inner surface (MTLA-FIS) were also prepared as control samples. Magnetic control of MTLA-SIS with engineered pore windows for liquid transport was operated similarly as mentioned above.

**1.6 Liquid residue test**

In order to observe the liquid residue, 20 μL water droplet containing rhodamine B (RhB, 1 mM) was used for the magnetic liquid transport by the actuators. Then, a part of the actuators was cut off, and the inner surface of the sample was characterized with confocal laser scanning microscopy (Figure 2h, Figure S5).

**1.7 Observation of the liquid film formed on the pore structure**

Nile red-loaded olive oil was used as the lubricant. The pore (~0.75 mm in diameter) was observed with CLSM before and after the water droplet (~20 μL) transport across the pore under the control of an applied magnetic field. An *in-situ* observation of the liquid film formation on the pore was also performed when the water droplet passed across the pore. It was noted that gravity replaced the magnetic field to propel the water droplet to transport as magnetic field was not easily operated on the platform of the CLSM.

In another experiment, calcein-loaded water droplet was controlled to pass across the pore structure. Then, a piece of dust-free paper was manipulated to contact the formed liquid film on the pore, followed by characterization with CLSM (Figure S9). The excitation wavelength of 543 nm and 494 nm was used for the observation of Nile red and calcein, respectively.

**1.8 Transport liquid contamination test**

The transport water droplet propelled by MTLA-SIS was characterized by fluorescence spectrophotometer to confirm the contamination. MTLA-SIS infused with Nile red-loaded olive oil was prepared for the experiments. ~20 μL water droplet was driven by MTLA-SIS to move to the position of the pore window. Then a syringe was used to carefully take a small amount of the water sample through the pore window. The collected sample was detected with a fluorescence spectrophotometer by using 543 nm as the excitation light (Figure. S11a). Nile red-loaded olive oil (10^-6^ M) was also tested as a control.

In addition, the collected transport water and the lubricant were further observed with CLSM at the same time. The excitation wavelength utilized was 543 nm. The water sample exhibited no fluorescence while the control sample (i.e., Nile red olive oil) was red (Figure. S11b).

**1.9 Liquid leak test**

In order to observe that whether liquid (transport water or lubricant oil) leaked from the pore window, Nile red-loaded olive and calcein-loaded water were selected as the lubricant and transport liquid, respectively. After magnetic control of MTLA-SIS for the water droplet transport across the pore window, the longitudinal cross section of the inner surface and outer surface at the position of the pore window was observed by CLSM (Figure S14). Excitation wavelength of 494 nm and 543 nm was used respectively to observe the Nile red and calcein. The inner surface at the position of the pore window exhibited obvious red fluorescence while there was no red fluorescence (corresponding to Nile red) or green fluorescence (corresponding to calcein) at the outer surface, indicating that water and lubricant did not leak through the pore window.

**1.10 Magnetic liquid transport with physical/chemical regulation**

To showcase the physical regulation, a 20 μL water droplet within the olive-infused MTLA-SIS was magnetically guided to the pore window position. Then, a 10 μL rhodamine B (RhB, 1 x 10^-4^ mol/L) solution was injected to mix with the droplet through the pore window. After loading with RhB, the water droplet moved forward under the control of the applied magnetic field.

For chemical regulation demonstrate, a 20 μL FeCl_2_ solution within the olive-infused MTLA-SIS was magnetically transported to the pore window position. Then, about 10 μL H_2_O_2_ (30w%) was injected to react with FeCl_2_ within MTLA-SIS through the pore window. Following the reaction, the FeCl_2_ droplet was chemically modified and its color changed. Alternatively, a 20 μl methyl orange solution introduced within the olive-infused MTLA-SIS was magnetically maneuvered near the pore window. Subsequently, a dropper containing 37w% HCl was carefully positioned adjacent to the pore window. Through diffusion, HCl vapor permeated the pore and reacted with the liquid droplet, resulting in the color change.

**1.11 Magneto-control of the external electric circuit**

An olive-infused MTLA-SIS with three pairs of pores (~0.75 mm in diameter) at the side surfaces was prepared, and subsequently integrated with an external electric circuit equipped with three light-emitting diodes (LEDs). In positions corresponding to each pair of pores, the circuit was intentionally disconnected. Then, a 20 μL droplet of conductive liquid (e.g., [BMIm]NTf_2_ or 1.0 M NaCl solution) was introduced into the MTLA-SIS and magnetically controlled to move. As the droplet reached the location of any paired pore, it bridged the circuit gap, and thereby completing the circuit and activating the corresponding LED. In this way, 3 LED would be selectively switched on or off upon the magnetic control. The sophisticated control of LED was constructed similarly.

**1.12 Mechanism of Magnetic Liquid Transport by MTLA-SIS**

The mechanism of the controlled liquid transport is attributed to the synergistic cooperation of asymmetric structure-induced Laplace pressure gradient and lubricant layer-induced low resistance. As shown in Figure S1a, the magnetic field induces the deformation of PDMS tube, forming a wedgelike structure. Such an asymmetric geometry generates a capillary force to drive the liquid to move. As the water contact angle on the slippery surface is lower than 90°, the net capillary force points towards the narrow end. Two cases are considered and the corresponding simplified models are then built as illustrated in Figure S1a and b. The opening angle is denoted by *α*, the width of the liquid by *L*, the distance of the liquid from the apex by *x*, the CA of liquid on the inner surface by *θ*. Due to the negligible contact angle hysteresis, *θ* is used to represent the advancing angle *θ_a_* and the receding angle *θ_r_*. Different curvatures are formed at the two sides of the droplet due to asymmetric deformation, which are labeled as *R_1_* and *R_2_*. According to the geometric relationship,^[^*^16,20,35]^*

$\frac{1}{R_{1}}=\frac{\cos\left( \frac{\alpha}{2}-\theta\right)}{x\sin\frac{\alpha}{2}}$ (1)

$\frac{1}{R_{2}}=\frac{\cos\left( \frac{\alpha}{2}+\theta\right)}{\left( L+x \right)\sin\frac{\alpha}{2}}$ (2)

As the opening angle α is small, the Laplace pressure $\Delta p_{1 ,}\Delta p_{2}$ of the liquid can be calculated by Yong-Laplace equation:

$\Delta p_{1}=\frac{2\gamma\cos\left( \frac{\alpha}{2}-\theta\right)}{x\sin\frac{\alpha}{2}}\approx\frac{4\gamma\cos\theta}{\alpha x}$ (3)

$\Delta p_{2}=\frac{2\gamma\cos\left( \frac{\alpha}{2}+\theta\right)}{\left( L+x \right)\sin\frac{a}{2}}\approx\frac{4\gamma\cos\theta}{a\left( x+L \right)}$ (4)

the pressure difference scales as

$\Delta p_{1}-\Delta p_{2}\approx\frac{4\gamma L\cos\theta}{\alpha x\left( x+L \right)}$ (5)

*Case 1: L << x (Figure S1a)*

The cross-sectional area of the liquid column is about $\pi{(\frac{x}{2})}^{2}$. Therefore, the driving force is

$F_{d}\approx\pi\alpha\gamma_{ow}L\cos\theta$ (6)

$\gamma_{ow}$ is the interfacial tension between the water and oil lubricant. The viscous force resisting its motion is given by *F_η_*$\sim2\pi{r\gamma}_{ow}{Ca}^{2/3}$,^[40]^ where $r$ is the radius of the tube, *Ca* is the capillary number and is obtained by $\eta_{o}V/\gamma_{ow},$ and $\eta_{o}$ is the dynamic viscosity of the lubricant oil, *V* is the moving velocity of the droplet. The along-surface resistance force $F_{a}$due to contact angle hysteresis is negligible.

In the case of the balance of the driving force and the resistance force, the maximum velocity could be obtained：

$V\sim{(\frac{\alpha L}{2r})}^{\frac{3}{2}}\frac{\gamma_{ow}}{\eta_{o}}\left( \cos\theta\right)^{\frac{3}{2}}$ (7)

In this case, the moving velocity is linearly related with $\frac{\gamma_{ow}}{\eta_{o}}\left( \cos\theta\right)^{\frac{3}{2}}$. As $\alpha$ is very small (shown in Figure S1a), the velocity is in the range of tens of micrometers per second. As the liquid droplet moves very slowly, and is not the focus of the paper.

*Case 2: L >> x (Figure S1b)*

The driving force could be derived as:^[39]^

$F_{d}\approx\frac{1}{3x}\pi\alpha\gamma_{ow}L^{2}\cos\theta$ (8)

The viscous force resisting its motion is given by *F_η_*$\sim\pi\alpha L\gamma_{ow}{Ca}^{2/3}$. Balancing the driving force and the resistance force generates：

$V\sim{(\frac{L}{3x})}^{\frac{3}{2}}\frac{\gamma_{ow}}{\eta_{o}}\left( \cos\theta\right)^{\frac{3}{2}}$ (9)

In this case, the moving velocity is also linearly related with$\frac{\gamma_{ow}}{\eta_{o}}\left( \cos\theta\right)^{\frac{3}{2}}$. According to the equation (9), when the droplet approaches the narrow end (*x*→0) , the velocity diverges. In our experiments, we obtained the maximum moving velocity when the droplet keeps a properly close distance from the apex. With the further decease of *x*, the accumulation of oil at the rim of of the droplet generates significant increase of friction, which stops the droplet from moving. The obtained maximum velocities were ploted with $\frac{\gamma_{ow}}{\eta_{o}}\left( \cos\theta\right)^{\frac{3}{2}}$, and the linear relationship was expected.


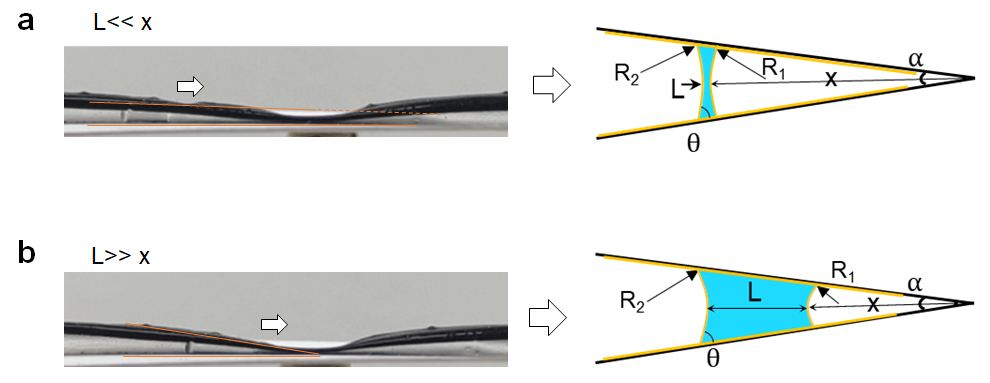


Scheme S1. Mechanism of magnetic-directed liquid transport. (a) Snapshot of a water droplet in the magnetic transport process in the case of the distance between the droplet and the apex is large (L << x). (b) Snapshot of a water droplet in the magnetic transport process in the case of the distance between the droplet and the apex is small (L >> x). A simplified model is correspondingly given in the figure.

Supplementary Figures


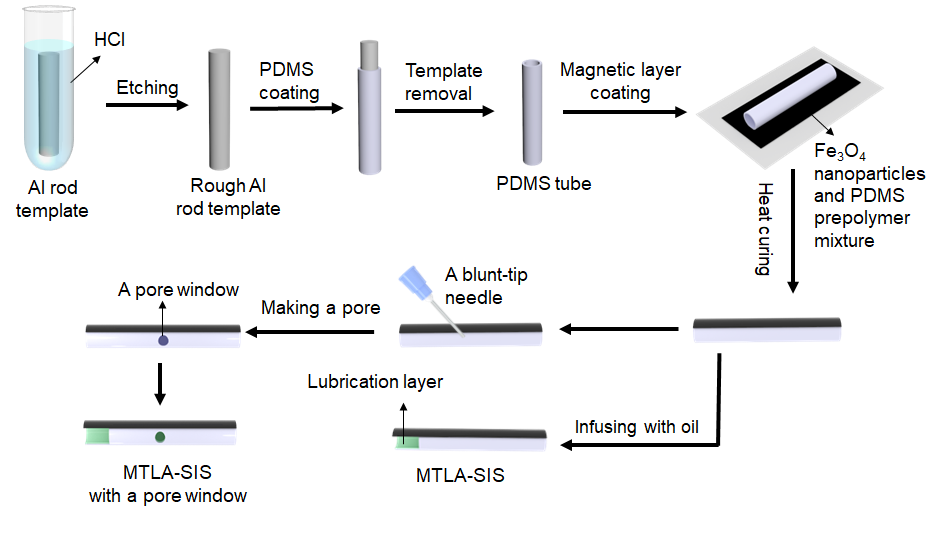


Figure S1. Schematic procedure for preparing magnetic tubular liquid actuators. Briefly, Al rod template was firstly etched by acid to form microstructures on the surface, followed by PDMS coating. After removal of the Al rod template, PDMS tube was obtained. Then one side of the outer surface of the PDMS tube was coated with magnetic layer (a mixture of iron oxide nanoparticles and PDMS prepolymer) by the contact method, followed by heat curing. A pore window structure could be made at the top or side surface of the tubular actuator on demand by using a blunt-tip needle as a puncher. The inner surface of the magnetic PDMS tube with/without pore windows was infused with lubricant, and magnetic tubular liquid actuator (MTLA-SIS) was finally obtained.


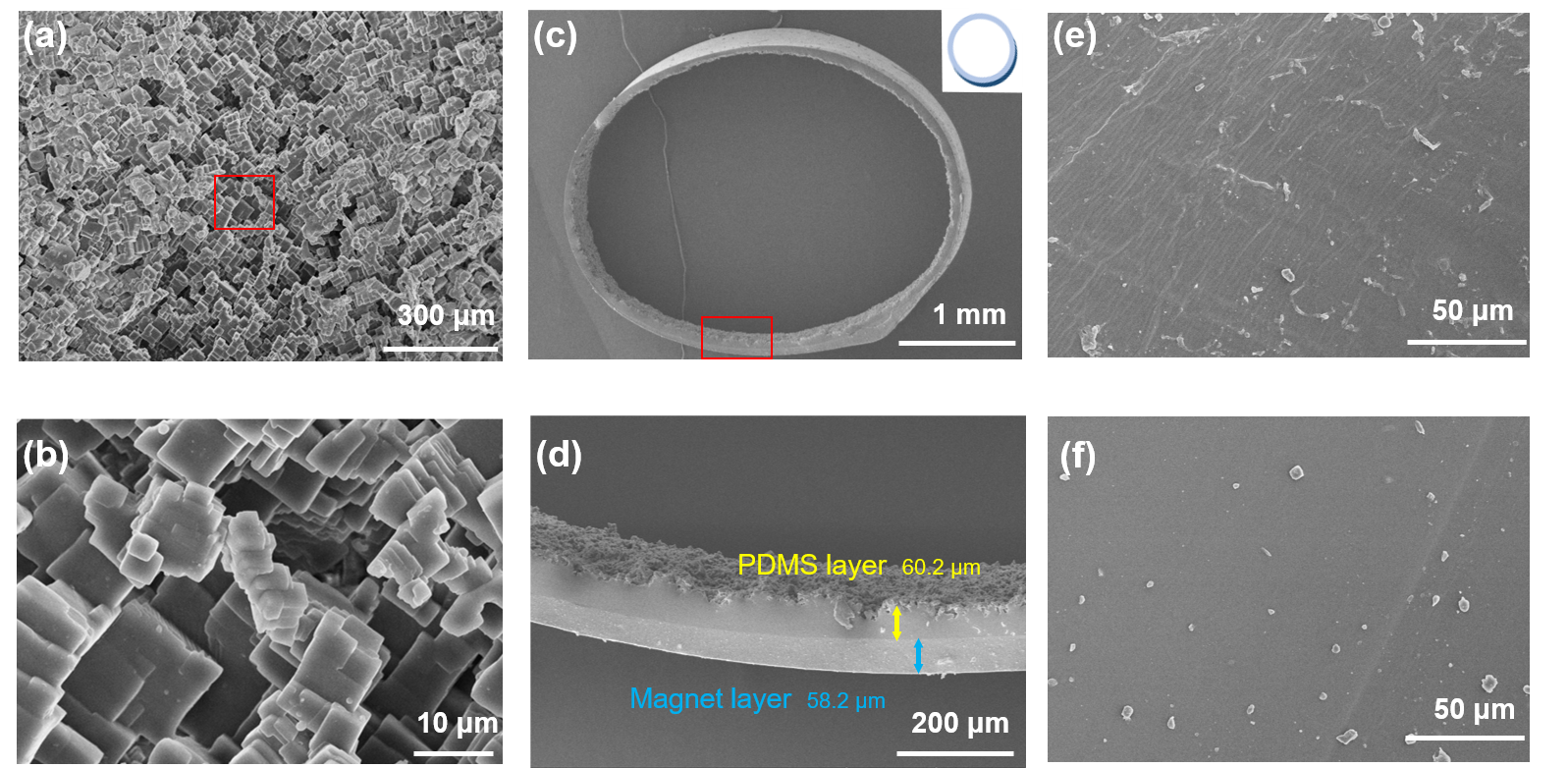


Figure S2. Microstructures of as-prepared tubular PDMS. (a) SEM image of the inner surface of the magnetic tubular PDMS obtained from rough Al rod template. (b) SEM image of the area marked in (a). (c) SEM image of the cross section of the magnetic tubular PDMS obtained from rough Al rod template. The inset is a schematic indicating the structure of the PDMS tube sample. The black part is the magnetic layer. (d) SEM image of the marked area in (c). (e) SEM image of the inner surface of the tubular PDMS demolded from Al rod without acid treatment. (f) SEM image of the PDMS surface obtained from a flat Petri dish template.


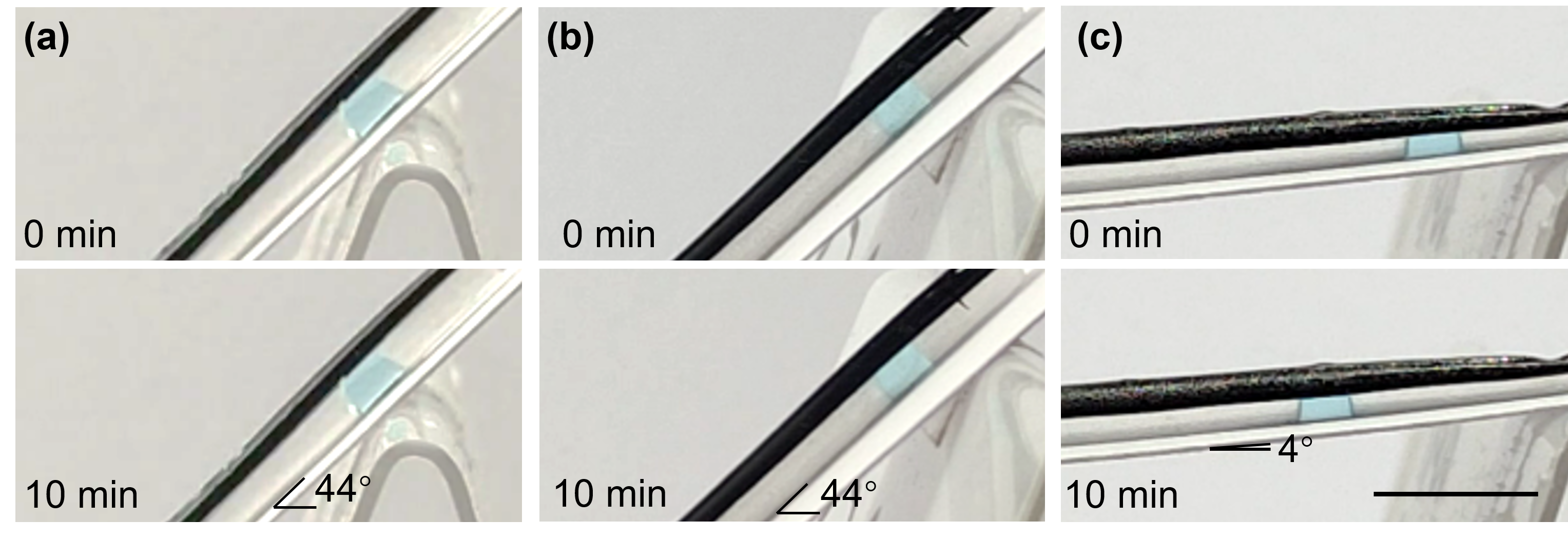
Figure S3. Gravity-driven liquid droplet motion in different magnetic tubular PDMS. (a) magnetic tubular liquid actuator with flat inner surface (MTLA-FIS), (b) magnetic tubular liquid actuator with rough inner surface (MTLA-RIS), (c) magnetic tubular liquid actuator with slippery inner surface (MTLA-SIS, infused with liquid paraffin). Water droplet in the tubular PDMS in (a) and (b) could not move even though the tubes were titled by 44°. In contrast, the droplet in the (c) could slide easily when the tube was titled by 4°. The scale bar is 1.0 cm.


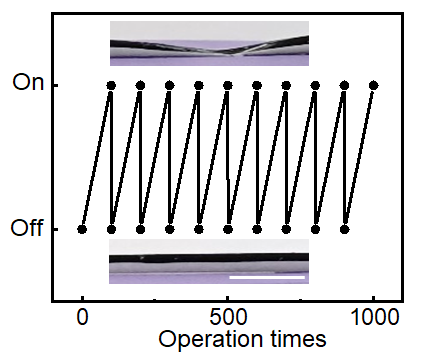


**Figure S4.** Mechanical stability test of MTLA-SIS after magnetic deformation and recovery. After 1000 times of cyclic operation, no obvious change was observed. The deformation of the actuator under magnetic field was defined as “On” state while the recovery of the actuator was “Off” state. The scale bar is 1.0 cm.

**
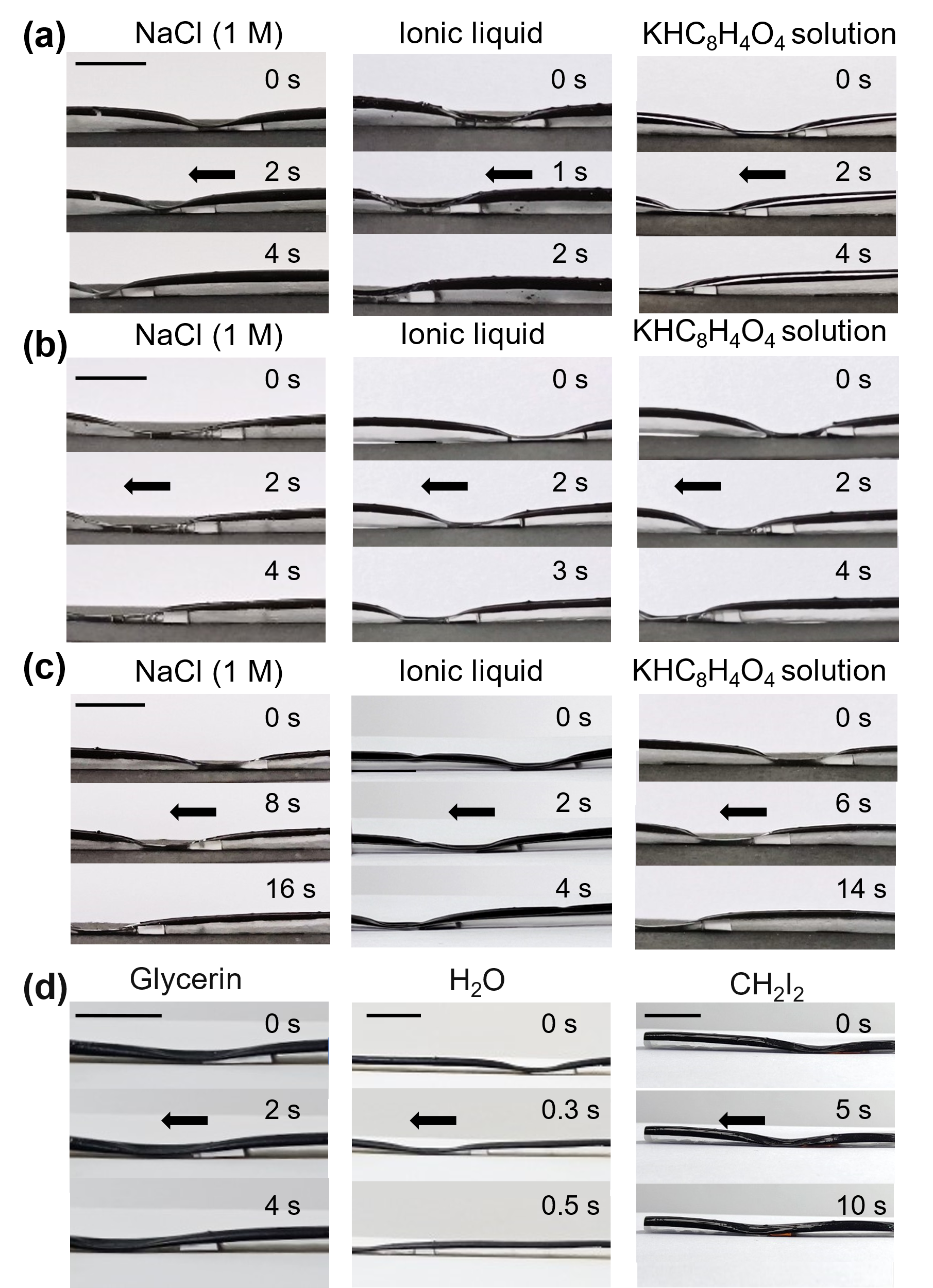
Figure S5.** Magnetic control of various liquid transport by MTLA-SIS infused with different lubricants. (a) Liquid paraffin-infused MTLA-SIS for magnetic transport of NaCl solution (1.0 M), [BMIm] NTf_2_, potassium hydrogen phthalate solution (KHC_8_H_4_O_4_, pH~4). (b) Silicon oil-infused MTLA-SIS for magnetic transport of NaCl (1.0 M) solution, [BMIm]NTf_2_, KHC_8_H_4_O_4_ solution (pH~4). (c) Olive oil-infused MTLA-SIS for magnetic transport of NaCl (1.0 M) solution, [BMIm] NTf_2_, KHC_8_H_4_O_4_ solution (pH~4). (d) [BMIm]NTf_2_-infused MTLA-SIS for magnetic transport of glycerin, water and CH_2_I_2_. The scale bar is 1 cm.


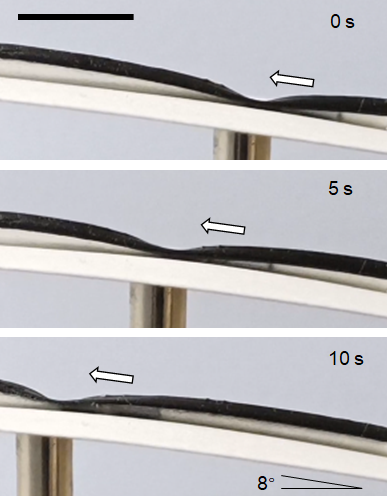


**Figure S6.** Magnetic control of water transport by MTLA-SIS (inner surface infused with olive oil) along an 8° incline. The scale bar is 1.0 cm.


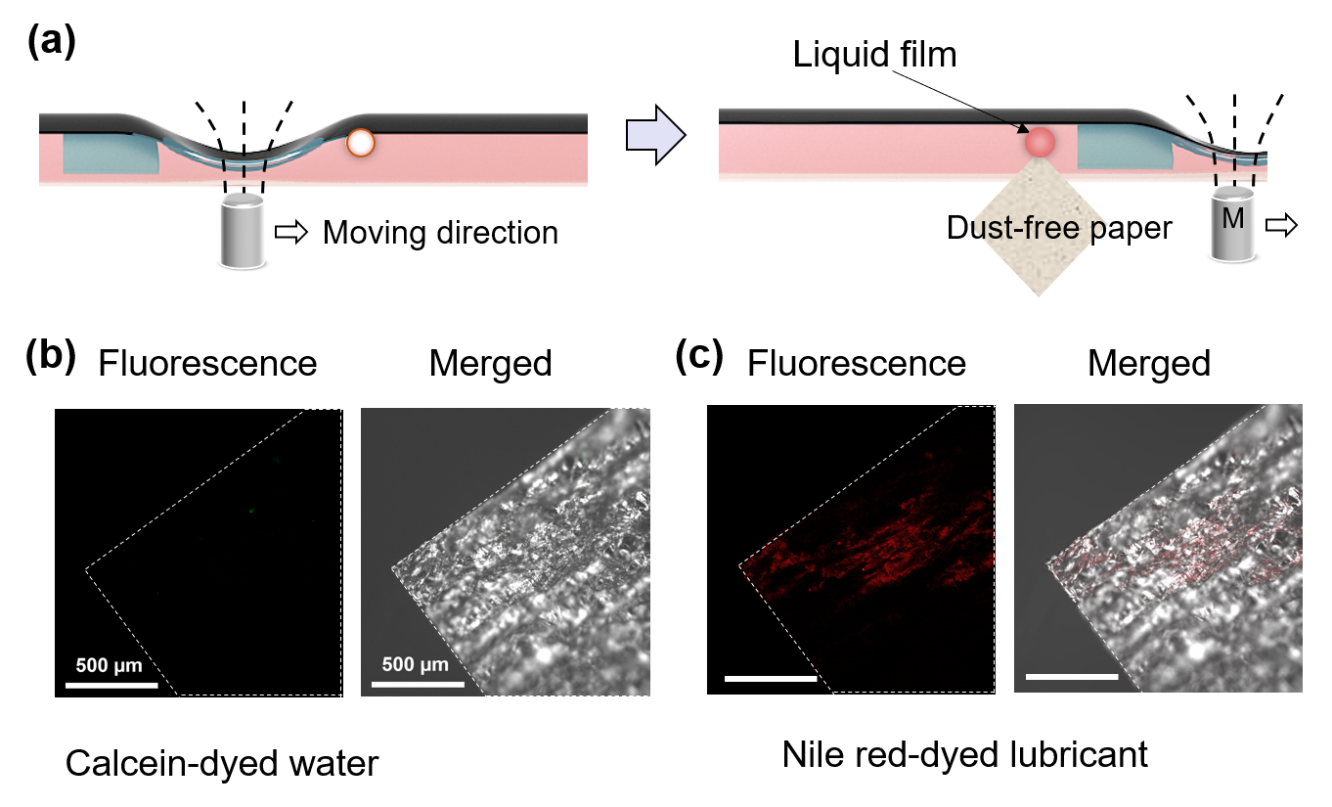


**Figure S7.** Liquid film confirmation by confocal laser scanning microscopy. (a) Schematic of magnetic transport of a calcein-dyed water droplet across the side pore window of MTLA-SIS infused with Nile red-dyed olive oil. A liquid film was formed on the pore and then a piece of dust-free paper was manipulated to contact the liquid film, followed by characterization with CLSM. (b) The confocal fluorescence (left) and merged (right) images of the dust-free paper. The excitation wavelength of 494 nm was used to detect the presence of calcein. (c) The confocal fluorescence (left) and merged (right) images of the dust-free paper. The excitation wavelength of 559 nm was used to detect the presence of Nile red. Obvious red fluorescence was observed while green fluorescence was negligible on surface of the dust-free paper, indicating the liquid film was formed by Nile red-dyed lubricant.


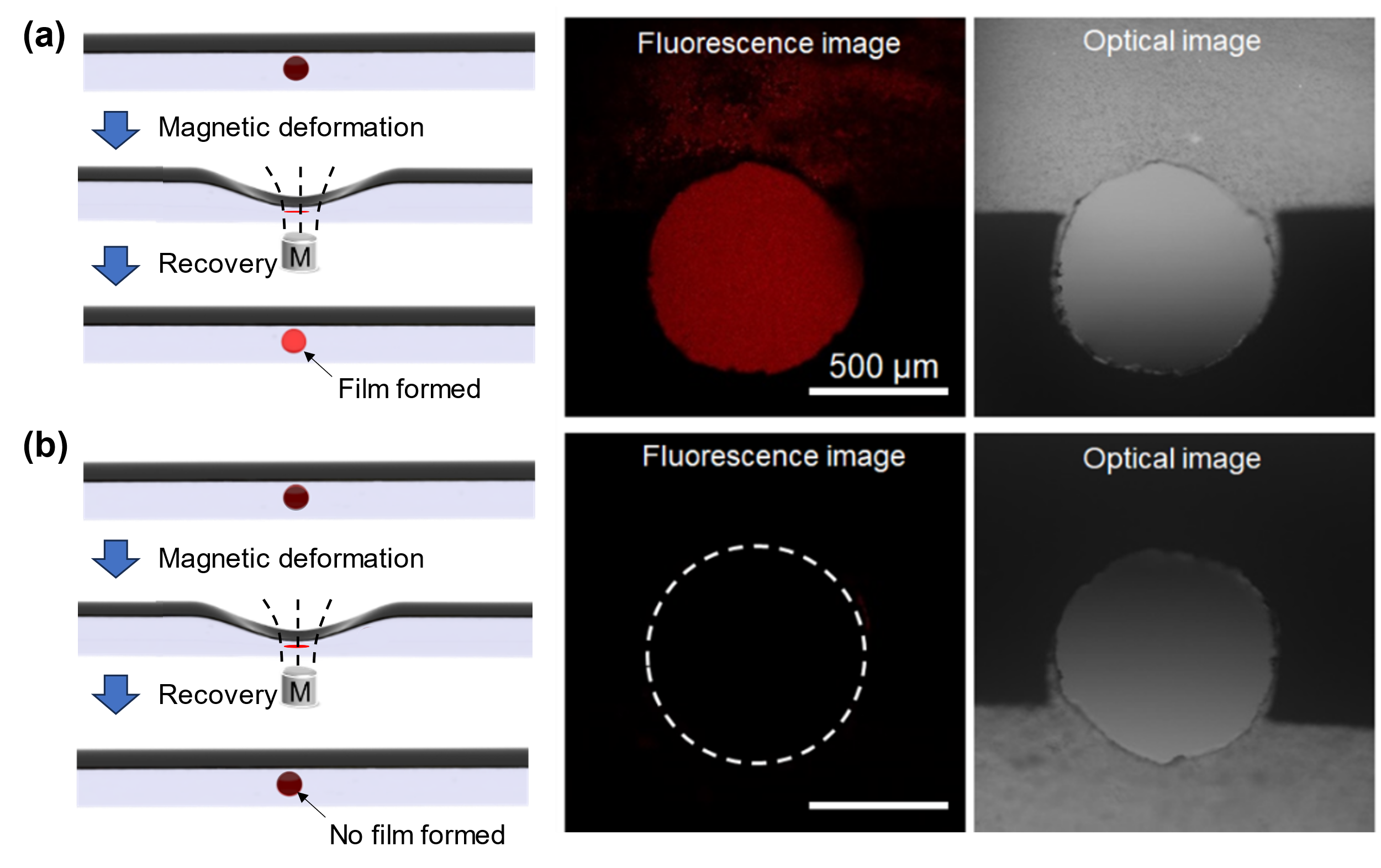


**Figure S8.** Liquid film formation on the pore structure depending on the thickness of the lubricant layer. (a) lubricant layer was 3.0 mg/cm^2^; (b) lubricant layer was 2.0 mg/cm^2^. MTLA-SIS infused with Nile red-dyed olive oil was magnetically controlled to deform and then recover at the pore position, which was immediately observed by CLSM, respectively.


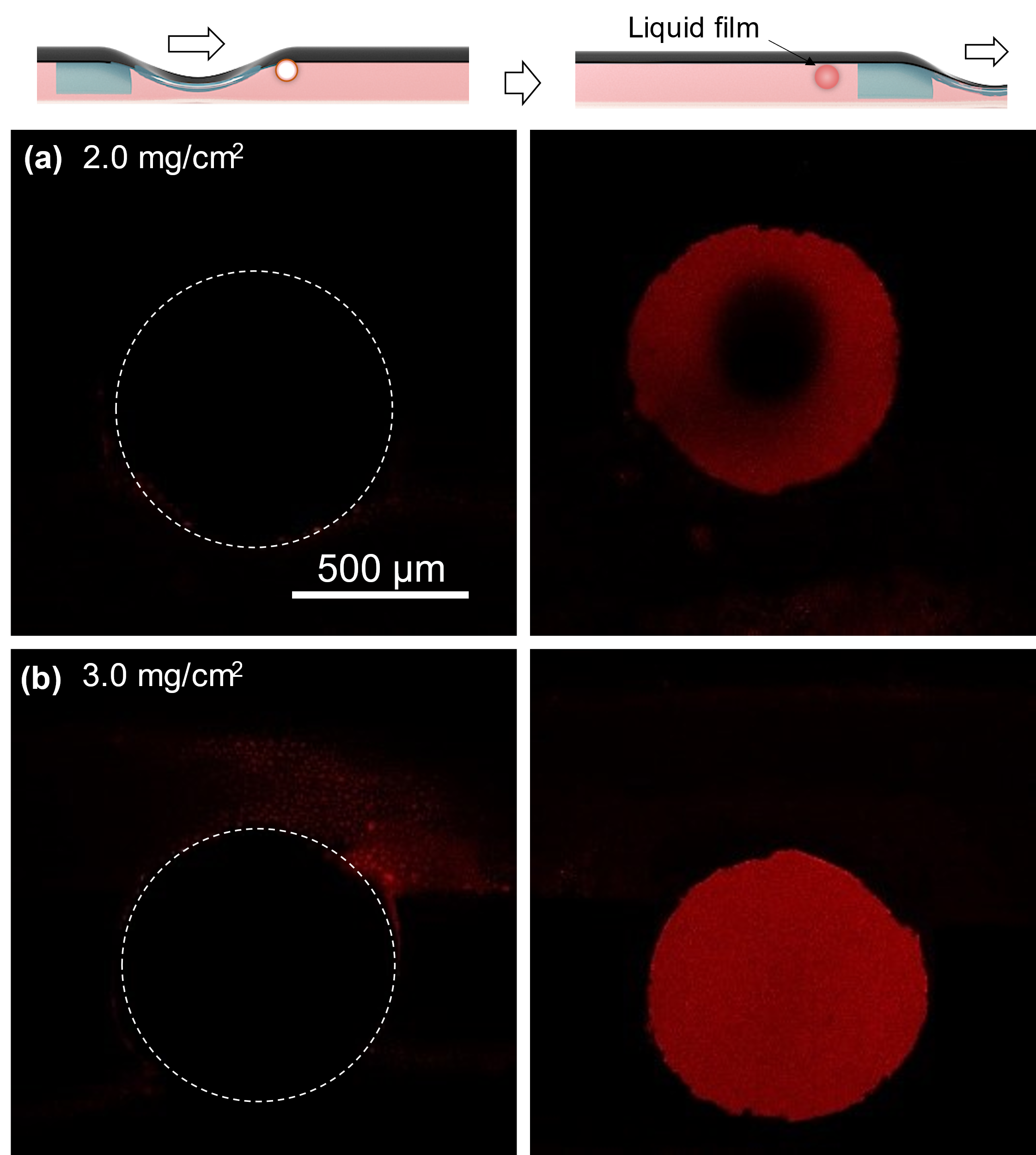


**Figure S9.** Liquid film formation on the pore structure after liquid droplet passing under the condition of different thicknesses of the lubricant layer. (a) The lubricant was 2.0 mg/cm^2^. (b) The lubricant was 3.0 mg/cm^2^. MTLA-SIS infused with Nile red-dyed olive oil was magnetically controlled to propel a water droplet to transport across the pore window, and then the sample was transferred to the CLSM for observation. In both cases, liquid film with red fluorescence formed on the pore. But the liquid film was very thin and the fluorescence in the center part was hardly observed in the case of (a). In addition, the liquid film in (a) was not stable, and ruptured in 10-30 s while it was relatively stable in (b).


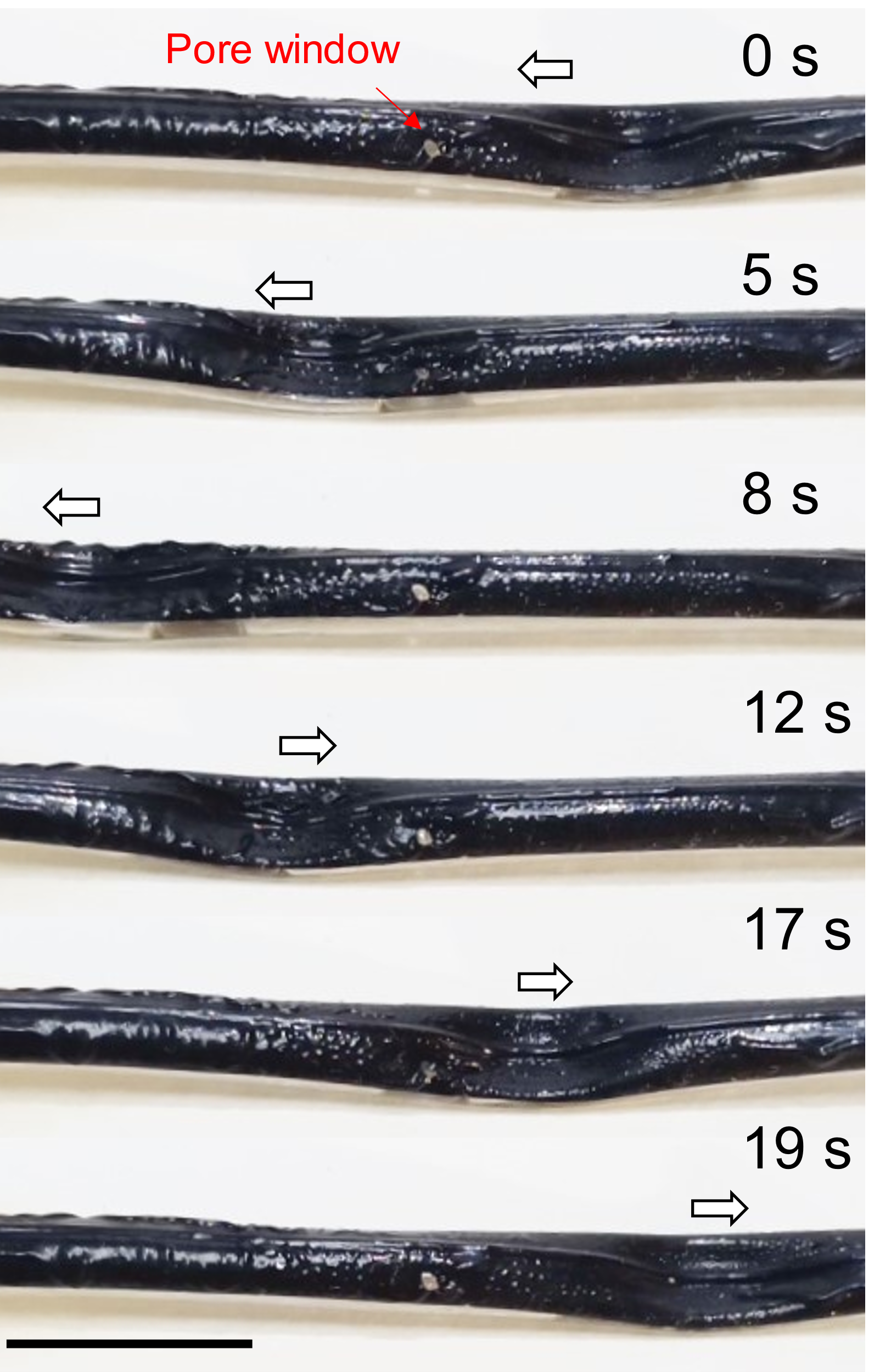


**Figure S10.** MTLA-SIS infused with olive oil for magnetic transport of water (20 μL) from right to left and then from left to right. The scale bar is 1.0 cm.

**
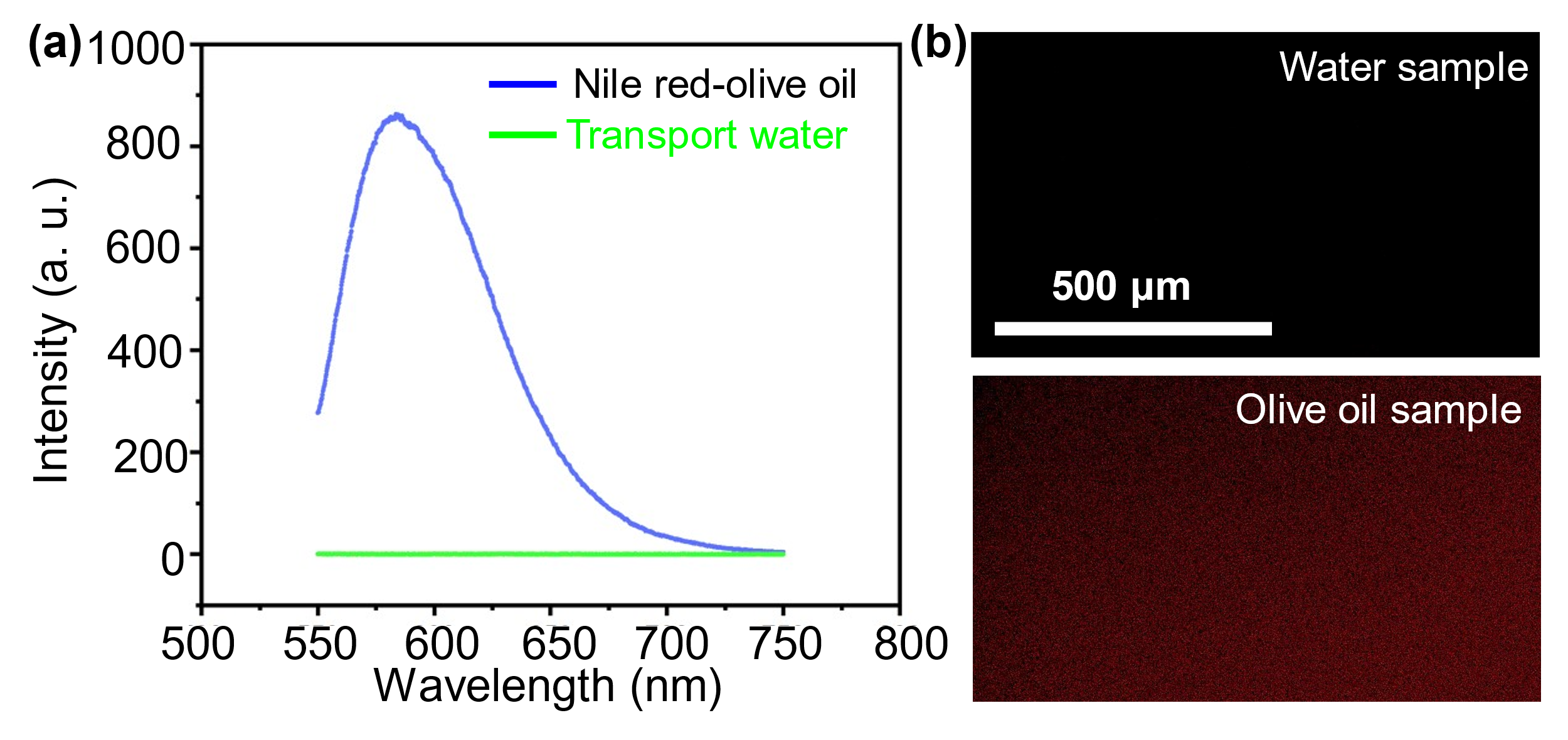
**

**Figure S11.** Contamination test of the transport water droplet by fluorescence spectroscopy. (a) Fluorescence spectroscopy of Nile red-loaded olive oil (as the lubricant, 10^-6^ M), and the collected transport water sample. (b) The confocal fluorescence images of the collected water sample (upper panel) and the corresponding Nile red-loaded lubricants (bottom panel). The collected water samples exhibit no fluorescence, indicating the transport water was not contaminated by the Nile red-loaded lubricants. The excitation wavelength is 543 nm.

**
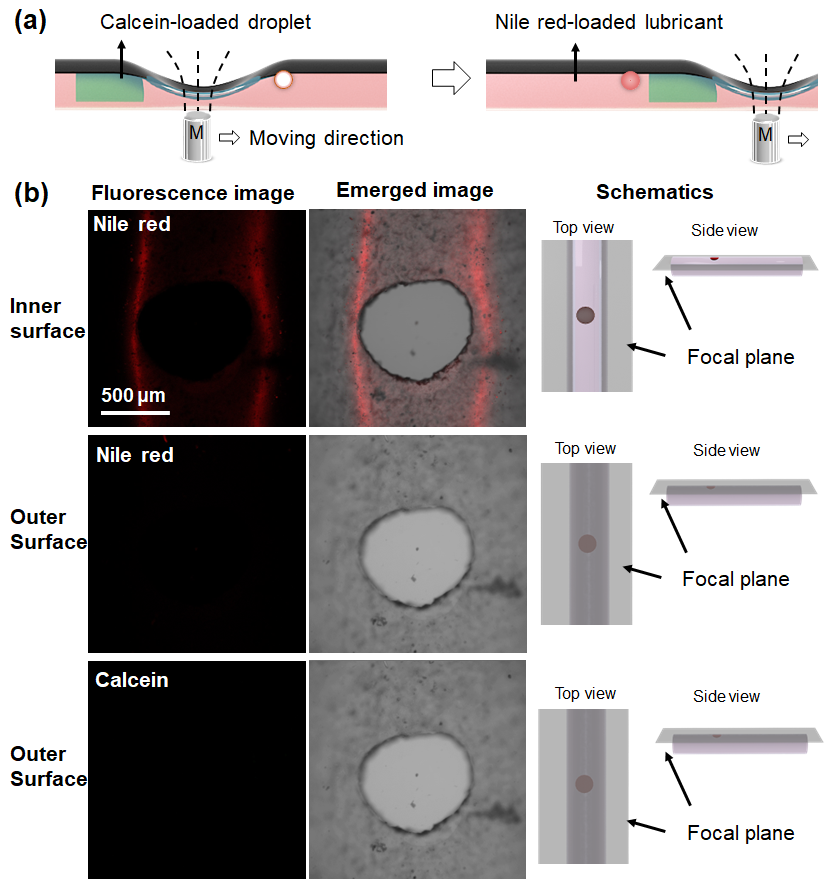
**

**Figure S12.** Liquid leak test by confocal laser scanning microscopy. (a) Schematic of magnetic transport of calcein-loaded water droplet by MTLA-SIS (infused with olive oil) with a side pore window. MTLA-SIS was infused with Nile red-loaded liquid paraffin. (b) Observation of the longitudinal cross section of the inner and outer surface at the position of the pore window. The excitation wavelength for Nile red and calcei was 543 nm and 494 nm, respectively.


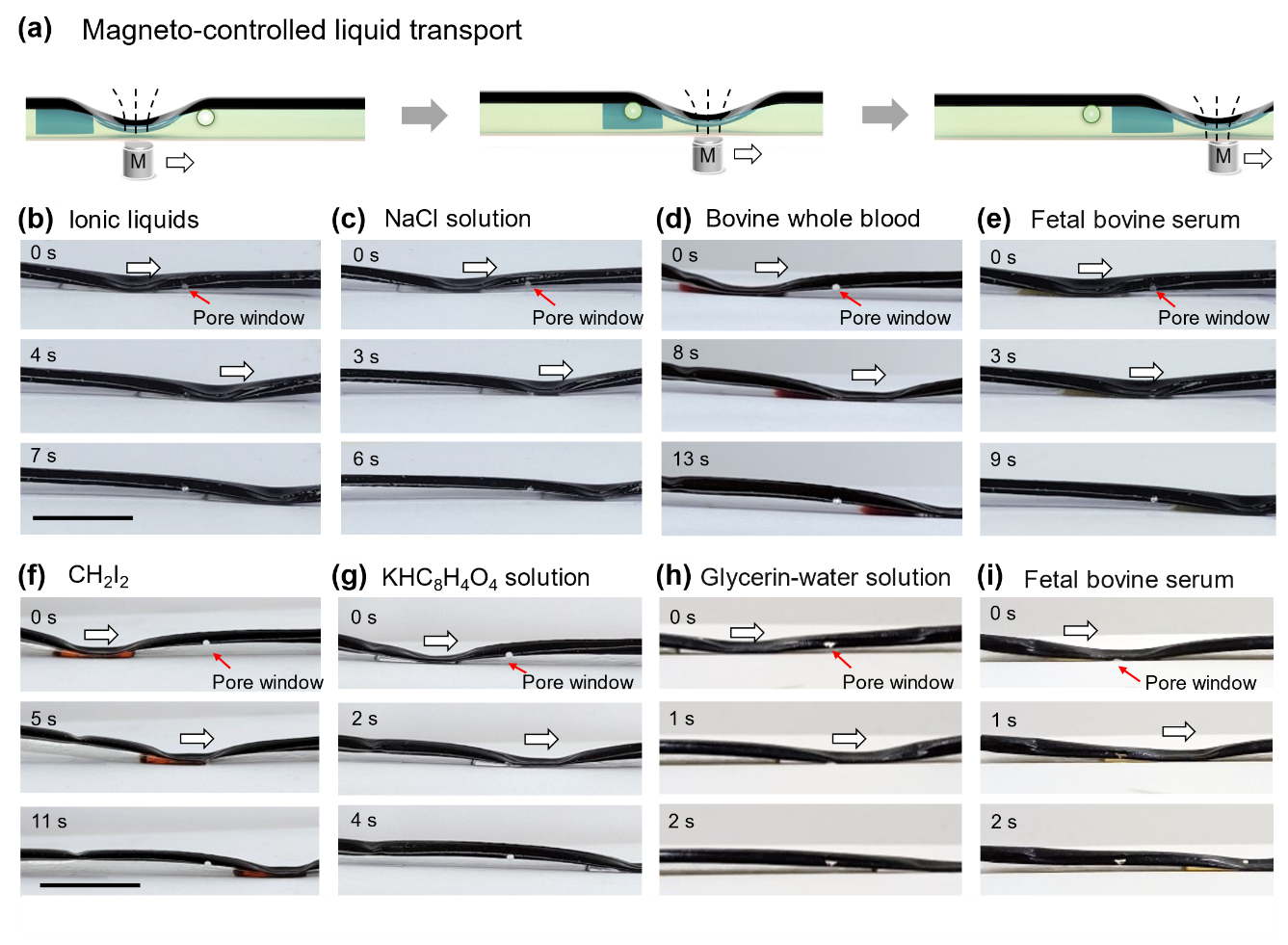


**Figure S13.** Magnetic-control of MTLA-SIS for directional transport of various liquids. (a) Schematic illustration of the process of magneto-controlled liquid transport. (b-e) Optical images showing MTLA-SIS infused with olive oil for magnetic transport of ionic liquid ([BMIm]NTf_2_) (b), and water-soluble liquids, including NaCl solution (1.0 M) (c), bovine whole blood (d), and fetal bovine serum (e). (f-i) Optical images showing MTLA-SIS infused with ionic liquid for magnetic transport of organic liquid CH_2_I_2_ (f), and aqueous solutions, including (KHC_8_H_4_O_4_ buffer solution (pH~4) (g), (glycerin-water mixture (volume ratio 1:1) (h), and fetal bovine serum (i). The scale bar is 1.0 cm.


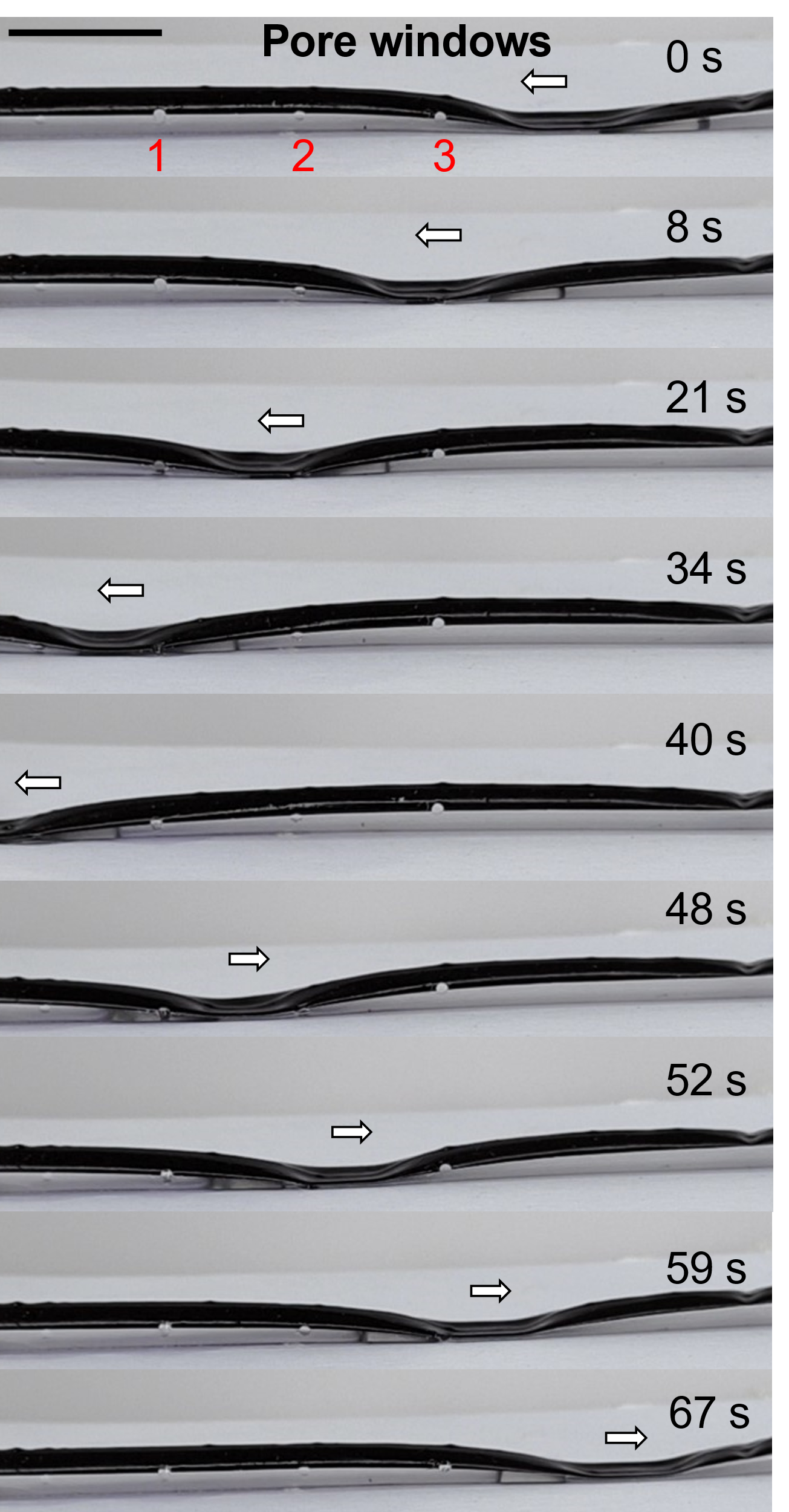


**Figure S14.** Time-lapse images of a MTLA-SIS with three pore windows for magnetic transport of a water droplet (~20 μL). The MTLA-SIS was infused with olive oil, and a water droplet was magnetically controlled to move across the pore windows sequentially from right to left and then from left to right. The scale bar is 1.0 cm.

Table S1. Properties of three lubricants or through laboratory testing.

|  | **Density, ρ  (g/cm^3^)** | **γ_o_**^[37]^  **(mN/m）** | **Viscosity, η (mPa·s, 25℃)** | **γ_ow_**  **(mN/m)** |
| --- | --- | --- | --- | --- |
| Liquid paraffin | 0.9 | 30 | 47.2 | 12.6 |
| Silicon oil | 0.96 | 21 | 145.5 | 50.2 |
| Olive oil | 0.91 | 32 | 85.4 | 19.9 |
| [BMIm]NTf_2_ | 1.44 | 33 | 50.0 | 13.6 |

Note: γ_o_ is liquid surface tension, and γ_ow_ is the interfacial surface tension between water and the liquid.

**Table S2.** Magnetic manipulation of conductive liquid droplets for sophisticated control of external circuits. Two magnetic tubular actuators, each featuring two pairs of through-hole pores in each actuator (labeled A, B, C and D), and were assembled and integrated with the external electric circuits. Specifically, pores A and B were connected in series with the left LED while pores C and D were connected in parallel with the right LED. The movement of conductive droplets was used to maneuver the pores, thereby establishing connections within the external circuits. A pore was designed as *“1”* When it was actuated by the droplet to make a connection; otherwise, it was *“0”*. Similarly, an LED was considered *“1”* when lit and *“0”* when off*.* The table presents a total of 16 possible outcomes.


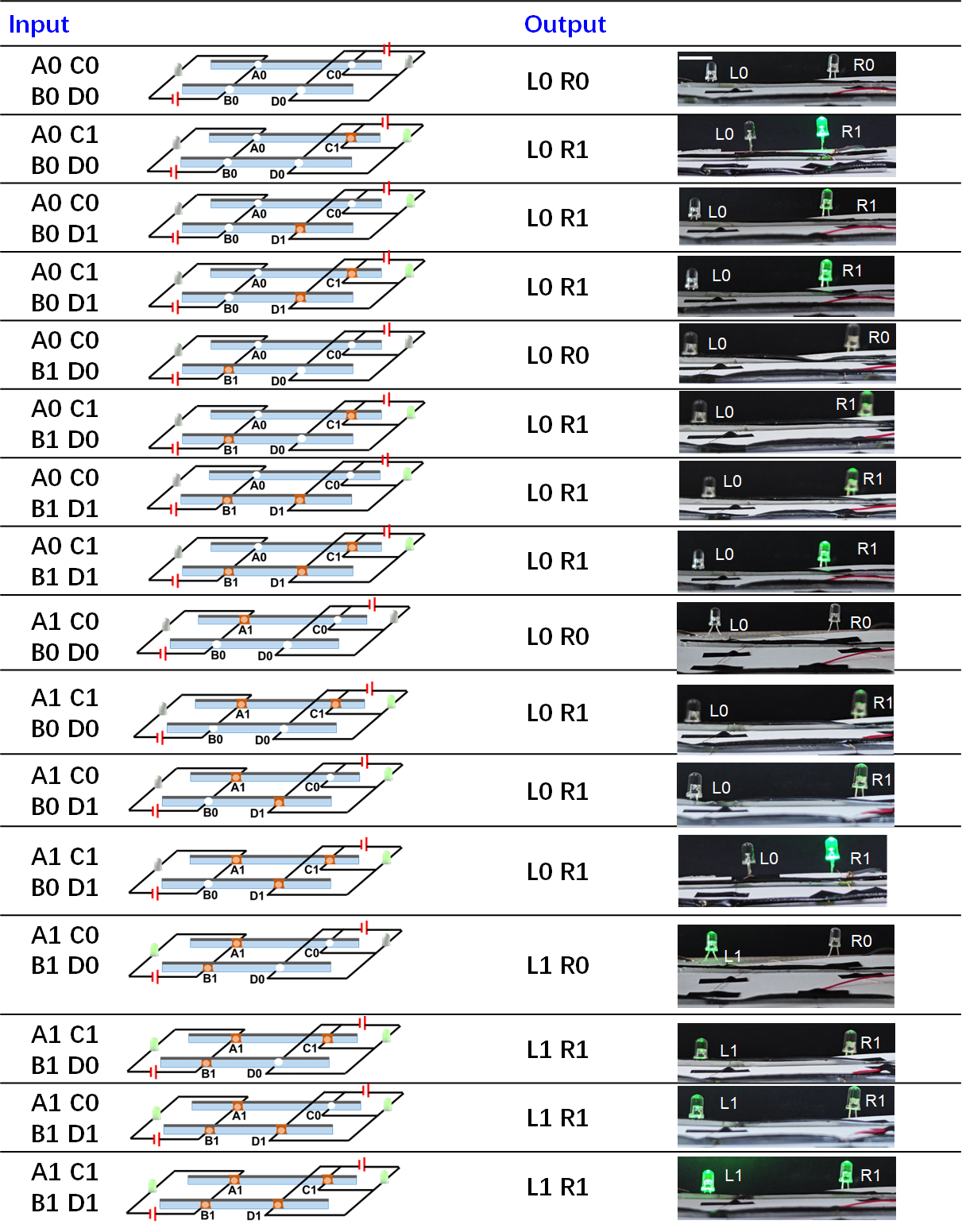

Supplement: Supplementary file 1 — Supporting Information [file ADVS-11-2406325-s005.docx]
